# A Mismatch EndoNuclease Array-Based Methodology (MENA) for Identifying Known SNPs or Novel Point Mutations

**DOI:** 10.3390/microarrays5020007

**Published:** 2016-04-05

**Authors:** Josep M. Comeron, Jordan Reed, Matthew Christie, Julia S. Jacobs, Jason Dierdorff, Daniel F. Eberl, J. Robert Manak

**Affiliations:** 1 Department of Biology, University of Iowa, Iowa City, IA 52242, USA; jordanreed.uiowa@gmail.com (J.R.); matthew.c.christie@gmail.com (M.C.); julie-jacobs@uiowa.edu (J.S.J.); jason-dierdorff@uiowa.edu (J.D.); daniel-eberl@uiowa.edu (D.F.E.); 2 Graduate Program in Genetics, University of Iowa, Iowa City, IA 52242, USA; 3 Department of Pediatrics, University of Iowa, Iowa City, IA, 52242, USA

**Keywords:** microarray, mismatch, endonuclease, SNP detection, genetic variation, disease mutation

## Abstract

Accurate and rapid identification or confirmation of single nucleotide polymorphisms (SNPs), point mutations and other human genomic variation facilitates understanding the genetic basis of disease. We have developed a new methodology (called MENA (Mismatch EndoNuclease Array)) pairing DNA mismatch endonuclease enzymology with tiling microarray hybridization in order to genotype both known point mutations (such as SNPs) as well as identify previously undiscovered point mutations and small indels. We show that our assay can rapidly genotype known SNPs in a human genomic DNA sample with 99% accuracy, in addition to identifying novel point mutations and small indels with a false discovery rate as low as 10%. Our technology provides a platform for a variety of applications, including: (1) genotyping known SNPs as well as confirming newly discovered SNPs from whole genome sequencing analyses; (2) identifying novel point mutations and indels in any genomic region from any organism for which genome sequence information is available; and (3) screening panels of genes associated with particular diseases and disorders in patient samples to identify causative mutations. As a proof of principle for using MENA to discover novel mutations, we report identification of a novel allele of the *beethoven* (*btv*) gene in *Drosophila*, which encodes a ciliary cytoplasmic dynein motor protein important for auditory mechanosensation.

## 1. Introduction

Current methodologies to identify known single nucleotide polymorphisms (SNPs) include microarray analysis (either based on differential hybridization strategies or hybridization coupled with DNA polymerase activity, as in the Affymetrix (Santa Clara, CA, USA) and Illumina (San Diego, CA, USA) platforms, respectively), Taqman^®^ assays (Roche Diagnostics, Indianapolis, IN, USA), MassARRAY^®^/iPLEX^®^ (Agena Bioscience, San Diego, CA, USA) single-base extension after amplification, mismatch endonuclease-based detection followed by suitable separation methods, or DNA sequencing (either directed or whole-genome, as in medical resequencing or next generation sequencing strategies) [1,2,3,4,5,6,7,8,9]. However, the aforementioned microarray-based, Taqman^®^, MassARRAY^®^ and mismatch endonuclease strategies are only able to interrogate SNPs whose identity is already known. Moreover, most of these methods are not easily scalable to query thousands of SNPs at a time with high accuracy. For instance, the mismatch endonuclease-based detection approach involves PCR to amplify target DNA fragments from both mutant and wild-type reference DNA, enzyme treatment to cleave hybrid heteroduplexes, and detection of potential variants using conventional gel electrophoresis or high-performance liquid chromatography (HPLC). On the other hand, direct sequencing of large genomic regions can identify novel SNPs (and other genomic variation) but oftentimes this strategy is time-consuming and can be expensive and inefficient when genotyping known variants.

We thus wanted to develop an economic methodology that could effectively identify both types of mutations (novel and previously discovered). Therefore, we combined the enzymatic efficiency and specificity of the mismatch endonuclease strategy with a tiling microarray hybridization strategy to produce a platform, which we call Mismatch EndoNuclease Array (MENA). In particular, we focused on the Surveyor^®^ endonuclease CEL II which is part of the CEL nuclease family derived from celery. CEL II specifically targets heteroduplex DNA (mismatched base pairs and small indels) and produces a double-strand cleavage at the mismatch site [8,10,11,12,13].

**Figure 1:**
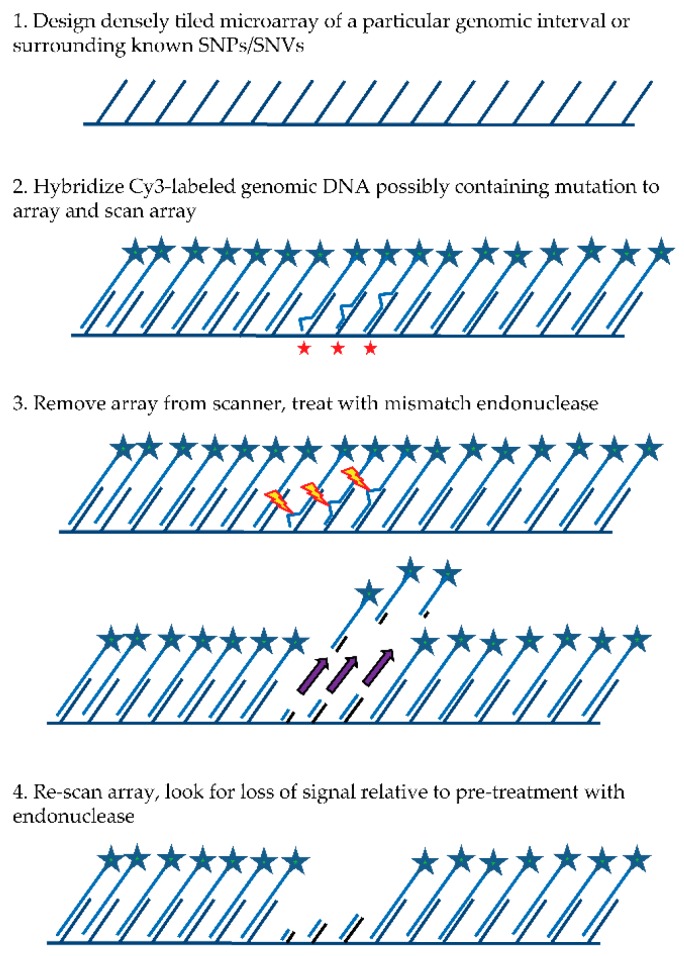
General Mismatch EndoNuclease Array (MENA) strategy. Red stars indicate probes with mismatches to the hybridized DNA. Lightning bolts represent mismatches where the endonuclease cleaves the duplex DNA.

The general MENA strategy (see Figure 1) relies on: (1) hybridizing a Cy3-labeled genomic DNA sample to a tiling microarray that interrogates one or more large genomic regions of interest in a sequenced genome; (2) scanning all features on the array to quantify hybridized DNA samples; (3) treating the array with a mismatch endonuclease (Surveyor^®^) that scans for and cleaves mismatches in heteroduplex DNA, reducing the length of heteroduplex formed by genomic fragments and array features when mismatches exist; and (4) re-scanning the array to obtain signal intensities post-treatment. Intensities of the post-digestion features are then compared with the intensities of the pre-digestion features. Probes with hybridized DNA including mismatches (SNPs and/or small indels) are expected to show a specific drop in post-treatment signal relative to pre-treatment signal that is more significant than for probes with no mismatches. It is important to note that traditional SNP array methods rely on a subtle reduction in hybridization strength (and thus signal) between DNA fragments from the sample and array probes when mismatches are present relative to perfect match. In contrast, MENA is expected to generate much more pronounced reductions in signal because genomic fragments are end-labeled and Surveyor^®^ treatment will shorten the heteroduplex via double-strand cleavage (acting on the labeled DNA sample as well as on the array feature or probe) and thus liberate signal from the array. Array features exhibiting the greatest relative drop in signal can validate the presence of a known SNP or, alternatively, identify a new candidate genetic variant that can be earmarked for validation using PCR and Sanger sequencing.

Here, we report 99% accuracy of the MENA platform in calling previously identified SNPs, as well as successful identification of novel, unknown genetic variation such as single nucleotide variants and indels. Using MENA, we also identify a novel mutation in a dynein motor protein gene required for hearing in *Drosophila melanogaster*, a mutation that was previously missed using standard Sanger sequencing. MENA is thus an effective alternative methodology for mapping unknown mutations localized to a genomic interval. Finally, given the rise in whole genome sequencing efforts, oftentimes with low sequence read coverage, we envision that this platform could play a significant role in SNP confirmation for these studies.

## 2. Materials and Methods

### 2.1. Array Designs

All designs were devised for the Roche NimbleGen 385K microarray. Design files (ndf and pos) for each array are available on request. Design strategies and genomic resources are articulated in the Results section.

### 2.2. Protocol for Labeling DNA, MENA Array Processing

One microgram of genomic DNA was labeled with Cy3-coupled random primers as described in the NimbleGen Arrays User’s Guide-CGH Analysis (version 5.1) using the labeling protocol for 385K feature arrays. Sixty-four micrograms of labeled DNA was lyophilized, followed by resuspension in the hybridization buffer including alignment oligo. Sixteen microliters was loaded into the array mixer (the chamber device that is affixed to the array in which hybridization of the labeled DNA occurs), and the sample was hybridized for up to 3 days.

The array was washed in 42 °C Wash Buffer 1 for 2 min, followed by Wash buffer 2 for 1 min, and wash buffer 3 for 15 s. The array was then dried for 15–20 s on an ArrayIt slide dryer. Arrays were scanned on either the Roche NimbleGen MS 200 microarray scanner set to auto gain, or the Molecular Devices Axon 2.5 uM resolution microarray scanner, and the PMT value was recorded.

Next, a new array mixer was placed on the dried, scanned array, and the array was placed on the NimbleGen Hybridization System set to 42 °C. Eighteen microliters of the Surveyor^®^ nuclease master mix (see below) was then added to the mixer, and the array was mixed for up to 40 min. The array was then rewashed at 42 °C in Wash Buffers 1 to 3 days as described above, and dried on the ArrayIt slide dryer. The array was rescanned with the same PMT setting as the first scan. Array data was then processed in NimbleScan as per the NimbleGen Arrays User’s Guide.

Surveyor^®^ nuclease reaction master mix was prepared, as following, using the relevant components of the Surveyor^®^ Mutation Detection Kit for Standard Gel Electrophoresis from Transgenomic, Inc. (now sold and registered by Integrated DNA Technologies (IDT), Inc., Coralville, IA, USA): 2.2 μL Surveyor^®^ Nuclease S, 2.2 μL Surveyor^®^ Enhancer S, 15.6 μL Surveyor^®^ Reaction Buffer (25 mM·Tris-HCl, pH 9.0; 50 mM·KCl; 10 mM·MgCl_2_). For further dilutions of Surveyor^®^, the Dilution Buffer used was as follows: 50 mM·Tris-HCl (pH 7.5), 100 mM·KCl, 0.01% Triton X-100, 10 uM·ZnCl_2_, 5% Glycerol.

### 2.3. Drosophila Strains

Flies carrying the *l(2)k07109* chromosome from the Kiss collection [14] contain a lethal PlacW insertion at polytene location 25F1-2, as well as an incomplete PlacW element that is *w*- at the *Fas3* gene locus in polytene section 36E. This chromosome fails to complement other *btv* alleles and deficiencies that define the *btv* locus [15]. We named this allele *btv^2^*. To maintain a non-lethal stock of *btv^2^*, we crossed the *l(2)k07109* chromosome to a deficiency, *Df(2L)cact255^rv64^*, leaving the *btv^2^* mutation hemizygous and the remainder of the chromosome freely recombining. We presume that over time, the deficiency has been lost, with the *btv^2^* allele becoming homozygous.

Because the background chromosome on which the Kiss lines were generated is not available, we used a different insertion line from the collection, *l(2)k12913* containing a PlacW insertion in the *rempA* locus [16], as a proxy for the genetic background. To recover this reference DNA, we used flies of genotype *rempA^k12913^/Df(2L)cact255^rv64^*.

## 3. Results

To test the feasibility of our strategy, we first designed a 385K Roche NimbleGen microarray that interrogates 128 human genome SNPs across 41 genes (see Supplemental Table S1). In this original design, the SNPs are interrogated by all four bases at the SNP position using four sets of oligonucleotides. Moreover, the SNP position region is tiled at 1 bp resolution, with oligonucleotides designed to position the SNP at every possible position along the oligo, plus 5 extra “buffer” probes on either side of each SNP that do not contain the SNP position. Thus, for a 30 m probe, 5 + 30 + 5 probes per strand and per SNP nucleotide are required for this design strategy, with 5 + 40 + 5 probes per strand per SNP nucleotide required for 40 mer probes, *etc.* Additionally, both forward and reverse strands are interrogated for each SNP region. This strategy was employed four different ways, using oligonucleotides that were 30, 40, 50 and 60 nucleotides long.

Our initial experiments demonstrated that only 60 m probes were able to perform as anticipated under the experimental conditions used (see Experimental Section), so we redesigned the array using only 60 m, and in addition to once again employing the overall design strategy outlined above, we made sure to include four replicates for each probe set in order to test whether increased replication might have an effect on call accuracy. Our MENA array, therefore, was designed to employ three out of the four probe sets (with either an A, G, C or T interrogating a particular SNP, using multiple replicates) to expose a mismatch, with only one set (with multiple replicates) being complementary to the SNP, thus protecting it from the Surveyor^®^ endonuclease. This strategy allowed us to test the efficacy of combining Surveyor^®^ and microarrays to correctly genotype specific nucleotide positions under a wide variety of conditions and different base pair mismatches. We also investigated the genotyping accuracy when different numbers of partially overlapping (tiling) probes are used to infer pre- *vs.* post-digestion signal in order to maximize sensitivity and reliability. For example, the 128 SNPs were further split into three groups of SNPs of similar size, one that was analyzed at 1 bp resolution, a second that was analyzed at 2 bp resolution, and a third that was analyzed at 3 bp resolution. Based on knowledge generated in our original set of arrays, all probes were designed to be 60 bp (see below).

**Figure 2:**
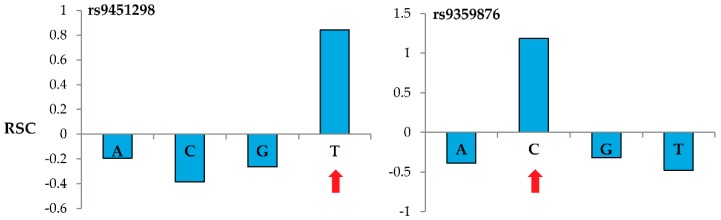
Relative signal change (RSC) between pre- and post-treatment arrays with Surveyor. Nucleotides (*X*-axis) indicate the nucleotides interrogated by the probes (not the nucleotides in the probe). In the sample analyzed, rs9451298 and rs9359876 have “T” and “C”, respectively (forward strand). See text for a detailed description of the RSC measure. Red arrows indicate identity of the SNP.

We performed several initial experiments using a previously genotyped CEPH (1362-02) human genomic DNA sample (Supplementary Table S1). We labeled the DNA with Cy3 using the standard Roche NimbleGen labeling protocol (see Experimental Section), and we varied both amount of DNA hybed, length of time hybed, and amount of Surveyor^®^ endonuclease used (Table 1). We then compared the pre- and post-digestion signals from the arrays to determine whether we could observe evidence of cleavage, and whether it was discriminate and showed specificity to heteroduplexes with mismatches. Once the pre and post digestion probe signal intensities were determined via the microarray scanner, we used an algorithm which we developed in house to reveal evidence of cleavage and assess whether MENA generated the correct SNP calls (accurate genotyping). We estimated the difference in relative signal due to Surveyor^®^ treatment by measuring the signal pre- and post-treatment for each of the four possible probes, where all nucleotides of the probe are the same except for a single site where each one contains a different nucleotide. Importantly, we normalized these changes to allow for differences in signal intensity among probes as well as overall reduction in signal after treatment and washing. Our measure of pre- and post-treatment change in signal intensity (“Relative Signal Change” or RSC) for each nucleotide (RSC*i* with *i* = A, C, G, or T) is expected to be positive for probes with a nucleotide with perfect match to the sample while all other probes with mismatched nucleotide are expected to generate negative RSC values, with the sum of all four RSC*i* equal to zero (see two examples in Figure 2). In detail,

(1) $$\text{RSC}i_{\left(i=A,C,G,T\right)}=\text{RS}i_{pre}-\text{RS}i_{post}=\;(Si_{pre}-{\bar{S}}_{pre})-(Si_{post}-{\bar{S}}_{post})$$

where pre- and post- indicate signal intensity pre- and post-treatment with Surveyor^®^, respectively, measured in *Log*_2_ units. S*i* indicates the signal for probes with nucleotide *i*, and $\bar{S}$ is the average signal for all four probes. Note that S*i* would represent the average signal among all replicate probes in an array. The probe with the most positive RSC*i* is therefore the probe corresponding to the perfect match and informs our genotyping at the interrogated nucleotide site. We classify the genotyping of a SNP as correct or accurate when the highest positive value among the four RSCi corresponds to the nucleotide with perfect match and correlates with the known SNP in the sample. Note that if the region is tiled densely enough, RSC*_i_* can be estimated from multiple adjacent probes, all of which interrogate the same SNP thus adding robustness to the study (see below).

Our analysis of several experimental and microarray conditions (Table 1) allowed us to discriminate cleavage and obtain high genotyping accuracy when using probes 60 nucleotides long. For 60-m probes, most experimental conditions allowed detection of a robust decrease in signal (negative RSC) for three out of the four sets of SNP-interrogating oligonucleotides while the fourth set showed a positive RSC, indicating that this latter set of probes contain a perfect match to the SNP present in the sample. Figure 2 shows representative results when genotyping two specific nucleotide sites using the MENA approach and 60-m probes. For these reference SNPs located in the BACH2 gene (rs9451298 and rs9359876), probes interrogating the correct nucleotide (T for rs9451298 and C for rs9359876) show a positive RSC while the probes interrogating the other three nucleotides show negative RSC values.

**Figure 3:**
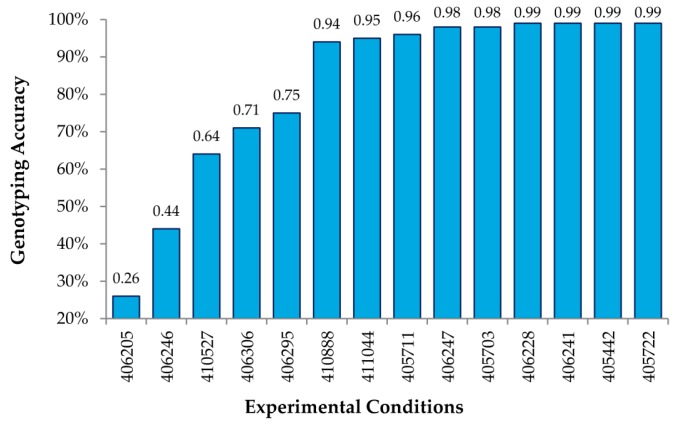
Summary of genotyping accuracy. Genotyping accuracy of MENA under different experimental conditions. Results shown after combining all homozygous SNPs for each condition. See Table 1 for detailed experimental conditions.

The analysis of many (up to 27) different combinations of experimental conditions allowed us to obtain a general set of guidelines to obtain high genotyping accuracy when using MENA. First, we determined that 2.2 μL of the Surveyor^®^ nuclease was optimal for achieving the maximal accuracy of calls (up to 99%) under a varied combination of amounts of DNA and digestion times (Table 1 and Figure 3). The use of limited (1.1 μL) Surveyor^®^ gave less accurate results particularly when digestion times were also reduced. Not surprisingly, the use of higher amounts of Surveyor^®^ (4.4, or 5.5 μL) required the reduction of digestion time (20 min instead of 40 min) to maintain high genotyping accuracy, likely to reduce unspecific cleavage. Either 40 or 50 min of digestion were able to achieve 99% accuracy when keeping all other parameters constant, including 64 μg of DNA hybridized, three days of hybridization and 2.2 μL of Surveyor^®^, suggesting that the enzyme digestion period could be somewhat flexible. Additionally, as little as 8 μg of amplified, labeled DNA (see Methods) could be used per hybridization to achieve this accuracy, using the same hybridization and digestion time, as well as Surveyor amount. Since labeling of the DNA usually results in an amplification of up to 70 fold, this suggests that as little as ~100 ng of starting genomic DNA might be sufficient to perform our analysis.

Figure 3 shows genotyping accuracy for known SNPs under 14 different experimental conditions, varying the amount of DNA hybed, concentration of the Surveyor^®^ endonuclease, and the time of digestion (Table 1). As shown, we obtained accuracies of 99% using the RSC algorithm described above for a number of conditions, thus indicating that MENA is not only highly accurate in genotyping SNPs, but also that the experimental conditions are fairly robust.

**Figure 4:**
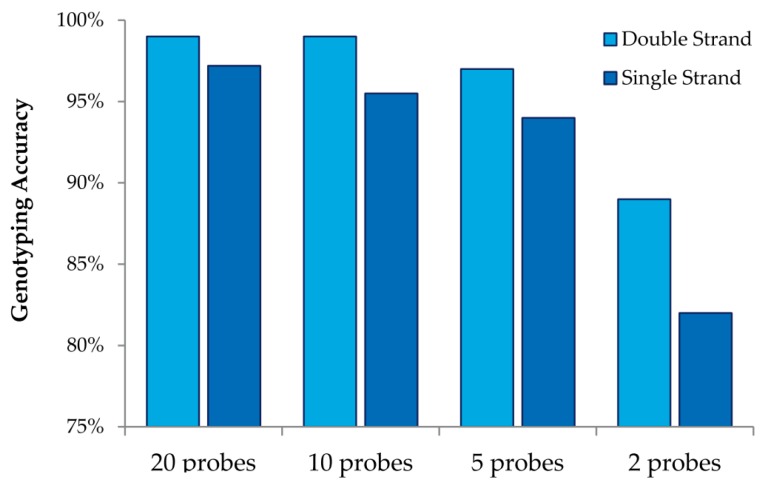
Genotyping accuracy with different number of probes. Percentage of genotyping accuracy based different number of probes interrogating a given SNP. Results shown after combining all homozygous SNPs under experimental conditions 405722 (see Table 1 and Figure 3). Because probes are 60 nucleotides long, analyses based on probes tiled every three nucleotides use the combined information of 20 probes, analyses based on probes tiled every six nucleotides use information of 10 probes, *etc*.

This study also allowed us to draw additional conclusions about array design when using MENA (Figure 4). We observed that interrogating both strands always increases genotyping accuracy relative to doubling the number of probes for a single strand. We also observed that high genotyping accuracy can be obtained with low tiling overlap for probes: 95% accuracy is obtained with only five overlapping probes (double strand and four replicates). Finally, we were able to investigate the accuracy in detecting different nucleotide mismatches. We observed that accurate genotyping was lowest (albeit still very high) for probes interrogating “T” variants (with accurate genotyping of 96%).

Having shown that MENA is highly efficient for detecting known SNPs, we next wanted to determine whether we could identify novel genomic variation. To this end, in our second array design we had also included the entire ~64 kb region of the human *IRF6* gene (including the intergenic space 5′ and 3′ to the gene; Chr1: 208,021,000–208,085,000, hg18), tiling both strands at 2 bp resolution, with two replicates of both strands. In this first attempt to investigate whether MENA could detect candidate novel SNPs in the IRF6 region, we used the 1362-1 CEPH sample and employed MENA under optimal conditions that were identified as indicated above. In this case, probes showing the largest signal reduction (signal change, SC) are candidates for harboring differences relative to the reference (array) genome. Note that for this study we did not include all four possible nucleotides at all possible sites across the entire IRF6 region. Instead, we applied a sliding-window approach using probes that match the reference genome to describe SC along the complete region analyzed, and focused on the probes showing the most extreme decay in signal (most negative SC). As a first approximation, we used a conservative FDR of 0.1% as threshold to identify putative probes harboring genetic variants in the sample analyzed.

When using the 1362-1 CEPH sample, we identified 18 genomic regions that had signal difference signatures consistent with the presence of a genetic change relative to the reference sequence. We designed primers to PCR the regions from the CEPH sample and performed Sanger sequencing in order to confirm the presence of novel mutations. We were able to identify 13 SNP variants (12 being heterozygous and one being homozygous; Supplemental Table S2) and indels in 10 of the interrogated regions. It is important to note that the identification of known SNPs is further validation that these variants are in fact real. Notably, we were able to identify not only homozygous single nucleotide mutations but also heterozygous mutations as well as indels.

**Figure 5:**
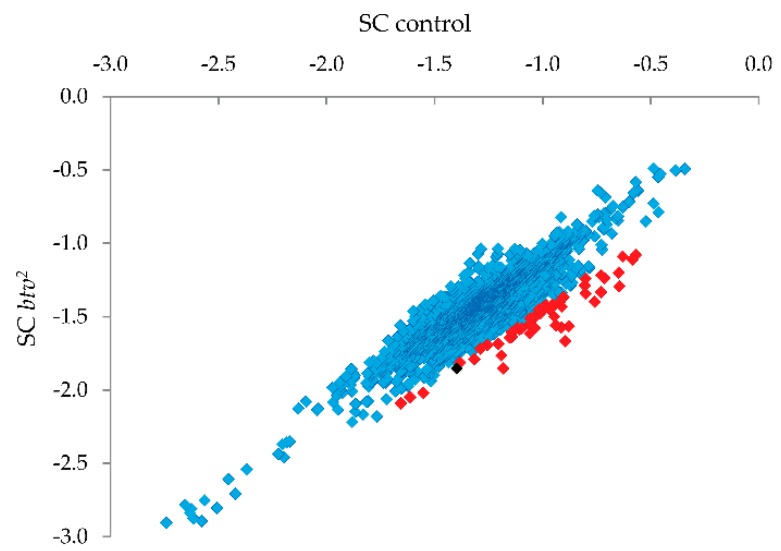
Comparison of signal change (SC) using MENA between mutant and control samples. SC indicates the reduction in signal after Surveyor treatment in controls and *btv^2^* samples. In red, probes showing the strongest (1%) reduction in signal in mutant *btv^2^* sample relative to controls and thus putative genetic variants. In black, the probe corresponding to a single bp deletion in exon 22 of the *btv* gene that results in a frameshift that causes early stop codons. In blue, all remaining probes that were interrogated with MENA. SC shown in *Log*_2_ units.

Finally, we wanted to determine whether we could identify an uncharacterized mutation from a model organism, *Drosophila melanogaster*. Previously, Eberl and colleagues identified a gene critical for fly hearing called *beethoven* (*btv*) [17,18]. Several mutations in this gene were identified; however, one allele that failed to complement the other *btv* alleles (*btv^2^*) was never characterized, as initial Sanger sequencing efforts covering several exonic regions based on early annotations failed to identify a likely mutation. The *btv^2^* mutation was discovered as a second-site lesion on a chromosome with a partial P-element insertion in the *fasciclin III* gene (*Fas3*) near *btv*. We thus considered the possibility that the *btv^2^* lesion resulted from a “hit-and-run” event during hybrid dysgenesis in the recovery of the *k07109* chromosome. To apply MENA we thus generated a tiling array design (OID34126) in which we tiled a portion of the *Drosophila* second chromosome (dm3, 2L: 17,933,592-18,006,679) using 60 m probes with 3 bp spacing (both strands, three total replicates per strand) and performed our MENA assay on DNA isolated from flies harboring the *btv^2^* allele (see Materials and Methods for a more detailed description) as well as flies that were wild-type for the *btv* locus. Note that, in this case, we used MENA with two samples (mutant and wild-type) and we compared the reduction in signal (SC) after Surveyor^®^ treatment for *btv* mutant samples (SC*_btv2_*) relative to the reduction in signal for the wild-type (SC_control_) sample. This approach, we believe, is superior to the one applied for IRF6 because can capture better differences among probes after Surveyor^®^ treatment. Probes showing the strongest relative reduction in the *btv^2^* sample (see Figure 5) were thus candidates for harboring uncharacterized SNPs or small indel variants.

**Figure 6:**
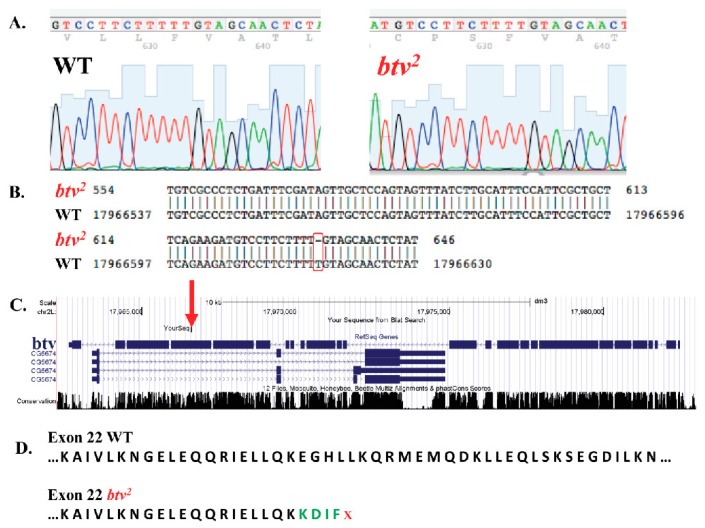
Identification of a novel allele of the *beethoven* (*btv*) gene in *Drosophila*. Results of PCR and Sanger sequencing around the candidate mutation detected by MENA confirm a single bp (“A”) deletion in *btv^2^* relative to a wild type (WT) sequence (**A**); this deletion maps to exon 22 of *btv* (red rectangle in **B**; red arrow in **C**), which is located in chromosome arm 2L (position 17966613-17966617); and (**D**) the consequences of the “A” deletion causing a frameshift and early stop codons. Green letters represent the altered amino acids that are translated as a result of the frameshift, which ends with a stop codon indicated by the red x.

Candidate SNP regions were then targeted for PCR and Sanger sequencing (Figure 5; red squares indicate the 1% of probes with strongest reduction relative to controls, and the black square is the probe amongst this 1% that led to identification of the relevant mutation), focusing on missense, nonsense or frameshift variants. Upon PCR amplification and sequencing of the candidate regions, a single base pair deletion mutation was confirmed in exon 22 (transcript *btv*-RD), at coordinate 17966613, which, on the minus strand encoding *btv*, begins a string of 5 T nucleotides that, in *btv^2^*, leave only four Ts. This indel results in a frameshift that causes early stop codons, thus substantially shortening the amino acid sequence of the cytoplasmic dynein protein (Figure 6). Thus, our MENA analysis succeeded in identifying a candidate novel variant that we experimentally confirmed to be a novel single base deletion mutation.

## 4. Discussion

We have developed a mutation detection system called MENA (Mismatch EndoNuclease Array) in which we couple mismatch endonuclease enzymology with tiling DNA microarrays. We believe this system will be particularly well-suited for verification of SNP and SNV calls for genomes sequenced at low coverage depths, whether they be from model organisms, commercially relevant organisms such as livestock or domesticated animals, or humans. Importantly, number of genomes sequenced is oftentimes considered more essential than achieving maximum accuracy in calling variants for each individual genome, as is often the case in large-scale human sequencing efforts. Although such a sequencing strategy allows the sequencing of more genomes, both low and high sequence coverage can invariably lead to inaccurate base calling (the latter due to systematic sources of error [19], particularly in species with large genomes and few novel variants, such as humans. Having an accurate and independent genomic strategy for calling the suspected variants will allow confirmation of their identity. The current Agilent 8-plex slide, which contains eight independent microarrays, for instance, would allow confirming more than 5000 variants per sample for less than $200, with the advantage that each array can be custom designed to include novel SNPs/SNVs and genotype eight different genomic samples per slide.

A second utility for MENA is identification of novel variants of interest in a genomic interval which can be tiled on a microarray. Such a strategy led us to identify a loss-of-function variant in the *beethoven* (*btv*) gene of *Drosophila melanogaster*, which is required for fly hearing [17,18]. Importantly, Sanger sequencing of a large genomic interval does not allow quick identification of relevant mutations, and the time required to design appropriate PCR primers, not to mention perform the sequencing, may be prohibitive depending on the size of the interval of interest. With MENA, regions of a genome that are up to approximately 50–100 kp can be assessed for variants in a matter of a few days once the arrays are synthesized. As shown, moreover, MENA can be applied to detect variants relative to a reference genome or to compare mutant and wild-type samples, and both approaches have allowed us to detect novel variants.

A third utility for MENA is the screening of panels of genes associated with a particular disease in order to quickly identify known or novel gene variants likely underlying the disease for a particular patient. This strategy could be particularly useful for diseases or disorders associated with large numbers of genes such as metabolic disorders. Moreover, since the newest array platforms contain large numbers of features (approximately one million features for the Agilent platform), a single array design could be used to interrogate disease genes from a number of different disorders, thus minimizing the number of array designs needed. After employing MENA, the researcher would only need to focus on analysis of the relevant genes for his or her disorder of interest.

We chose to use Roche NimbleGen microarrays during the planning phases of this study due to their long oligos, which provide increased sensitivity and specificity over short oligonucleotide microarrays. Although Roche NimbleGen no longer produces microarrays, Agilent microarrays have equivalent long oligonucleotides (similarly built base by base off the array surface) and oligo density. Thus, it is likely that our strategy will work for the Agilent platform as well. MENA, we propose, can be a rigorous, user-friendly and efficient methodology to simultaneously validate and test the presence of multiple known SNPs and/or detect novel variants using long-oligo array-based hybridization assays.

## 5. Conclusions

We have developed an accurate, efficient low-cost mutation detection methodology (Mismatch EndoNuclease Array, or MENA) that pairs DNA mismatch endonuclease enzymology with tiling microarray hybridization to detect novel or known genomic variation at the level of point mutations or indels, provided the genome or genomic region of interest has been sequenced. Importantly, MENA is an independent genomics methodology that can be used to confirm variants identified by next generation sequencing methodologies, especially in cases where sequencing coverage is low. Since correlating genomic variation with disease is a primary focus of the biomedical research community, new platforms that can aid in correctly characterizing such variation will be in demand.

## Figures and Tables

**Table 1:** Experimental conditions to study genotyping accuracy using MENA.

Slide Number	Amount of DNA (μg)	Amount of Surveyor (μL)	Time of Surveyor (min)
405722	64	2.2	50
405442	64	2.2	40
406241	16	2.2	40
406228	8	2.2	40
405703	64	4.4	20
406247	32	2.2	40
405711	64	5.5	20
411044	64	4.4	40
410888	64	2.2	30
406295	64	2.2	20
406306	64	1.1	50
410527	64	3.3	20
406246	64	1.1	40
406205	64	1.1	20

All MENA experiments allowed three days of hybridization. See text for details.

## Supplementary Materials

The following are available online at www.mdpi.com/2076-3905/5/2/7/s1.
