# Supplementary material for: A Mismatch EndoNuclease Array-Based Methodology (MENA) for Identifying Known SNPs or Novel Point Mutations
Source: Microarrays (Basel). 2016 Apr 5;5(2):7. doi: 10.3390/microarrays5020007 (PMC5003483; doi:10.3390/microarrays5020007)
Supplement: Supplementary file 1 [file microarrays-05-00007-s001.pdf]

Supplementary Materials: A Mismatch EndoNuclease Array-Based Methodology (MENA) for Identifying Known SNPs or Novel Point Mutations

Josep M. Comeron, Jordan Reed, Matthew Christie, Julia S. Jacobs, Jason Dierdorff, Daniel F. Eberl and John Robert Manak

Table S1. SNPs from 41 genes in the human genome.

Gene	rs Number	Chrom	Position	Genotype 1362-1 (NA10860)
ACY3	rs2252577	chr11	67174613	CC
BACH2	rs2236181	chr6	90718297	AG
BACH2	rs9451298	chr6	90717040	TT
BACH2	rs9353711	chr6	90759159	TT
BACH2	rs9359876	chr6	90701504	CC
BACH2	rs11969265	chr6	90739765	CT
BACH2	rs1065273	chr6	90695111	CC
BACH2	rs404256	chr6	90714504	TT
BACH2	rs7756942	chr6	90851291	GG
BACH2	rs207259	chr6	90872539	AG
BHMT	rs651852	chr5	78444816	AA
BHMT	rs3733890	chr5	78457715	AG
BHMT	rs506500	chr5	78450093	CT
BMP4	rs17563	chr14	53487272	TT
CRISPLD2	rs2326398	chr16	83460218	AG
CRISPLD2	rs4783099	chr16	83498830	CC
CRISPLD2	rs16974880	chr16	83500139	TT
DHFR	rs1643638	chr5	79966012	CT
EPHA7	rs535926	chr6	94164863	AG
EPHA7	rs4707796	chr6	94133139	TT
EPHA7	rs346462	chr6	94061206	AA
EPHA7	rs164532	chr6	94113634	AA
EPHA7	rs164290	chr6	94080416	TT
FAF1	rs11205760	chr1	50946918	CT
FAF1	rs1149795	chr1	50745152	TT
FAF1	rs3827730	chr1	50710436	CT
FAF1	rs2455636	chr1	50616760	GG
FGFR1	rs13317	chr8	38388671	CT
FGFR1	rs6996321	chr8	38441503	GG
FGFR1	rs881301	chr8	38451475	CT
FGFR1	rs10958704	chr8	38447459	GG
FGFR2	rs2981582	chr10	123342307	TT
FGFR2	rs3750817	chr10	123322567	CC
FGFR2	rs10749423	chr10	123395011	TT
FOLR1	rs2071010	chr11	71578612	AG
FOLR2	rs514933	chr11	71607855	AG
FOXE1	rs1443433	chr9	99619040	TT
FOXE1	rs1443432	chr9	99623016	AA
FOXE1	rs10217225	chr9	99636812	CC
FOXE1	rs894673	chr9	99652091	TT
FOXE1	rs3758249	chr9	99653961	GG
FOXE1	rs1867278	chr9	99655770	AA

FOXE1	rs1443434	chr9	99657300	TT
FOXE1	rs874004	chr9	99661939	CG
FOXE1	rs10984103	chr9	99679096	CC
GABBR2	rs967931	chr9	100445382	TT
GABBR2	rs570138	chr9	100507758	CT
GABRB3	rs1426217	chr15	24372218	TT
GABRB3	rs981778	chr15	24508333	AG
GABRB3	rs1432007	chr15	24361782	AG
GABRB3	rs2059574	chr15	24548136	AT
GABRB3	rs890317	chr15	24473294	AC
GAD1	rs3749034	chr2	171381721	CC
GAD1	rs2058725	chr2	171398367	AA
IRF6	rs126280	chr1	208086447	GG
IRF6	rs2235377	chr1	208042015	CT
IRF6	rs658860	chr1	208057172	TT
IRF6	rs4844880	chr1	207937539	TT
IRF6	rs2357231	chr1	208095077	GT
IRF6	rs685248	chr1	208074990	CG
IRF6	rs2235543	chr1	207927291	CC
IRF6	rs846908	chr1	207925076	GG
IRF6	rs713075	chr1	207830873	GG
IRF6	rs11808690	chr1	207958073	TT
IRF6	rs2272866	chr1	207996076	GG
IRF6	rs2236902	chr1	207947668	AA
IRF6	rs2064148	chr1	207976008	AA
IRF6	rs11807619	chr1	207947996	GG
IRF6	rs2013162	chr1	208035307	AC
IRF6	rs17317411	chr1	208027937	TT
IRF6	rs2235371	chr1	208030703	CT
IRF6	rs611861	chr1	208003874	CC
IRF6	rs2298930	chr1	207962835	CC
IRF6	rs2236898	chr1	207885175	GG
JAG2	rs10220575	chr14	104712530	TT
JAG2	rs3784240	chr14	104694306	CC
JAG2	rs2239282	chr14	104704544	CT
MSX1	rs12532	chr4	4916047	AA
MTHFD1	rs2236225	chr14	63978598	CT
MTHFD1	rs1950902	chr14	63952133	CC
MTHFR	rs1801131	chr1	11777063	CC
MTHFR	rs1801133	chr1	11778965	CC
MTR	rs1805087	chr1	235115123	AA
MTR	rs1806505	chr1	235063198	CT
MTRR	rs1532268	chr5	7931179	AA
NNMT	rs2301128	chr11	113673209	GG
PAK1	rs521417	chr6	10851958	CC
PDGFC	rs3815861	chr4	157903698	CT
PTCH1	rs2236407	chr9	97277617	AG
PTCH1	rs10512248	chr9	97299524	AA
PVRL1	rs11217415	chr11	119079819	TT
PVRL1	rs7129848	chr11	119071438	CC
PVRL1	rs4459318	chr11	119046988	CC
RDH10	rs7843902	chr8	74372941	GG

RDH10	rs17214921	chr8	74392900	CC
ROR2	rs6479383	chr9	93719176	CC
SATB2	rs994185	chr2	199850836	AG
SHH	rs167020	chr7	155312494	GG
SHH	rs6975820	chr7	155308437	GG
SNAI	rs11076696	chr16	87267489	GG
SNAI	rs6091080	chr20	48037294	GG
SNAI	rs1056707	chr16	87269005	CC
SNAI2	rs1992375	chr8	50000397	TT
SOX9	rs1042667	chr17	67632146	AC
SPRY2	rs504122	chr13	79809526	TT
TBX1	rs1978060	chr22	18129525	AG
TBX1	rs7293017	chr22	18113811	GG
TBX1	rs4819519	chr22	18116003	CT
TBX1	rs5993820	chr22	18117621	CT
TBX1	rs737869	chr22	18139437	CG
TBX1	rs2238778	chr1	18138399	GT
TBX10	rs2514022	chr11	67158689	CC
TGFA	rs2902345	chr2	70570107	CC
TGFA	rs377122	chr2	70620533	CC
TGFA	rs432203	chr2	70618196	GG
TGFA	rs454305	chr2	70589727	AA
TGFA	rs503314	chr2	70528257	GG
TGFB3	rs2268626	chr14	75514520	CT
TGFB3	rs2284792	chr14	75513332	AG
TGFB3	rs3917201	chr14	75499308	AG
TGFB3	rs3917210	chr14	75497533	AT
TGFB3	rs3917192	chr14	75501427	AG
TGFB3	rs11159163	chr14	75569255	CC
TGFB3	rs11159161	chr14	75539115	CT
TGFBR1	rs7874221	chr9	100908889	TT
TGFBR1	rs10760673	chr9	100918443	GG
TGFBR1	rs7042852	chr9	100942648	TT
TGFBR1	rs10988719	chr9	100935303	TT

Table S2. *IRF6* genotypes identified using MENA (CEPH sample 1362-1).

SNP	Reference	Genotype
rs2073485	G	G/A
rs2236907	C	C/A
rs2236908	G	G/C
rs2236909	A	A/G
rs11119346	C	C/T
rs11119347	T	T/C
rs2069068	G	G/T
rs11119348	A	A/C
rs596731	G	A
rs597673	C	C/G
rs3753516	G	G/C
rs7536857	T	T/C
rs10863792	G	G/A
